# Application of the Canadian C-Spine rule and nexus low criteria and results of cervical spine radiography in emergency condition

**DOI:** 10.11604/pamj.2018.30.157.13256

**Published:** 2018-06-21

**Authors:** William Ngatchou, Jeanne Beirnaert, Daniel Lemogoum, Cyril Bouland, Pierre Youatou, Ahmed Sabry Ramadan, Regis Sontou, Maimouna Bol Alima, Alain Plumaker, Virginie Guimfacq, Claude Bika, Pierre Mols

**Affiliations:** 1 Department of Emergency and Cardiac Surgery, St Pierre University Hospital, Université Libre de Bruxelles, Belgium; 2 Department of Emergency St Pierre University Hospital, Université Libre de Bruxelles, Belgium; 3 Department of Cardiology, Erasme University Hospital, Université Libre de Bruxelles, Belgium; 4 Department of Radiology, St Pierre University Hospital, Université Libre de Bruxelles, Belgium; 5 Department of Cardiac Surgery, St Luc University Hospital, Université Catholique de Louvain, Belgium; 6 Department of Cardiology, Ixelles University Hospital, Université Libre de Bruxelles, Belgium; 7 Université de Douala, Cameroun

**Keywords:** Cervical spine X-ray, emergency, quality

## Abstract

**Introduction:**

The Canadian C Spine Rule (CCR) and the National Emergency X-Radiography Utilization Study (Nexus) low criteria are well accepted as guide to help physician in case of cervical blunt trauma.

**Methods:**

We aimed to evaluate retrospectively the application of these recommendations in our emergency department. Secondly we analyzed the quality of cervical spine radiography (CSR) in an emergency setting.

**Results:**

281 patients with cervical blunt trauma were analyzed retrospectively. The CCR and the NEXUS rules were respected in 91.2% and 96.8% of cases respectively. No lesions were found in 96.4% of patient. A lesion was present in 1.1% of patient and suspected in 2.5% of patient. The quality of CSR was adequate in only 37.7% of patient. The poor quality of CSR was due either to the lack of C7 vertebrae visualization in 64.6% or other lower vertebrae in 28%. Other causes included the absence of open mouth view (8%), the absence C1 vertebrae visualization (3.4%), artifact in 2.3% and the absence of lateral view in 0.6% of patient.

**Conclusion:**

CCR and NEXUS are widely used in our emergency department. The high rate of inadequate CSR reinforces the debate about it’s utility in emergency condition.

## Introduction

**Figure 1:**
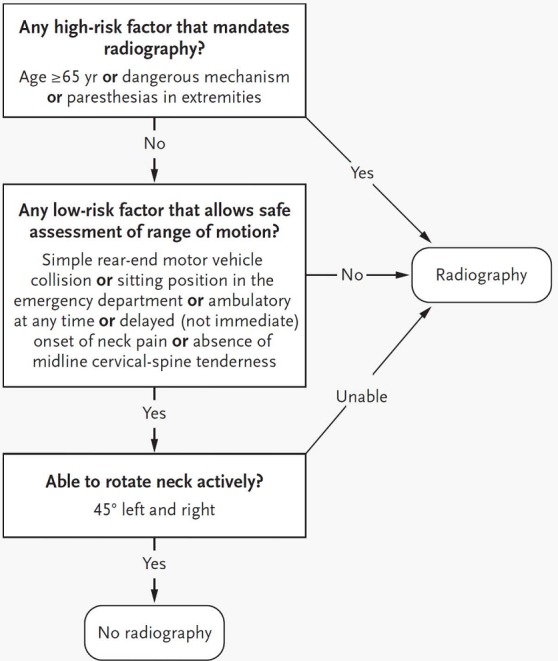
The Canadian C-Spine rule

Cervical blunt trauma is major health problem in developed countries [1,2]. Missing a cervical-spine fracture is an obsession of many emergency departments’ physicians, leading to unnecessary cervical-spine radiography (CSR). Two decision rules have been developed independently to permit more selective ordering of CSR, more rapid ruling out of injury to the cervical for patients, decrease patients’ exposure to ionizing radiation and economic losses [3]. In 1992, The National Emergency X-Radiography Utilization Study (NEXUS) Low-Risk Criteria (NLC) developed one simple decision making instrument based on five clinical criteria (Table 1) that can help physicians to identify reliably the patients who need CSR after blunt trauma [4,5]. The second decision rule was the Canadian Cervical-Spine Rule (CCR) developed in 2001 in ten Canadian emergency departments [6]. This rule uses 3 high-risk criteria (age 65 year or older, dangerous mechanism, paresthesias in the extremities), 5 low criteria (simple rear-end motor vehicle crash, sitting position in emergency department, ambulatory at any time, delayed onset of neck pain, and absence of midline C –spine tenderness), and the ability of patients to actively rotate their necks, to determine the need for CSR (Figure 1). While these rules are widely accepted, their current application and results are poorly studied. The aim of this study was to evaluate retrospectively the compliance of our emergency physicians to these recommendations. The second objective was to assess the quality of CSR performed in emergency settings.

**Table 1:** The NEXUS low-risk criteria

Cervical-spine radiography is indicated for patients with trauma unless they meet all of the following criteria
No posterior midline cervical-spine tenderness
No evidence of intoxication
A normal level of alertness
No focal neurologic deficit, and
No painful distracting injuries

## Methods

We retrospectively analyzed the file of all patients over 15 years old who underwent CSR following blunt trauma in our emergency department between January 2013 and December 2015. Patient with penetrating trauma and patient who underwent firstly Computer Tomography (CT) of the cervical spine were not included. For each eligible patient, data concerning mechanism of injury, the symptoms, and the respect of both NEXUS and CCR rules and the result of radiography were recorded. The quality of cervical CSR was defined by a senior radiologist as adequate (presence of anterior, lateral, and open mouth images, presence of all of seven cervical vertebrae, No artifact) or inadequate (absence of one or more than one vertebrae, absence of lateral or open mouth images, the presence of artifact). This protocol was approved by the local ethic committee (CHU St Pierre, Brussels, Belgium Agreement number: O.M. 007).

### Statistical analysis

Quantitative data were presented as mean ± standard deviation (SD) and qualitative data were presented as counts and frequencies. Means were compared between good and poor quality of radiography using Student-t test while frequencies were compared using Chi squared test. All analyses were performed using SPSS 2.0 software.

## Results

Overall 281 patients were included. Demographics and clinical characteristics are summarized in Table 2. The mean age was 38.6 years. The mechanism of trauma was high in 70.8% of cases, 40.6% presented local pain. CCR and Nexus rules were respected in 91.8% and 96.8% of cases respectively. No lesion was found in 96.4% of patient. A lesion was present in 1.1% of patient and suspected in 2.5% of patient. The quality of X-ray was good in only 37.7% of patient. The poor quality of X-ray was due to the absence C7 vertebrae in 64.6%, the absence of more than C7 vertebrae in 28%, the absence of open mouth imaging in 8%, the absence of C1 in 3.4%, artifact in 2.3% and no profile in 0.6% of patient (Table 3).

**Table 2:** Demographics and clinical characteristics

Variable	Modality	Patients	Frequency (%)
Sex	Women	131	46.6
	Men	150	53.4
Intensity	Low	82	29.2
	High	199	70.8
Complains of neck pain	No	114	40.6
	Yes	167	59.4
Distracting injury	No	97	34.5
	Yes	184	65.5
Hematoma/wound lesion	No	208	74.0
	Yes	73	26.0
Unconsciousness	No	263	93.6
	Yes	18	6.4
Paresthesias in extremities	No	264	94.0
	Yes	17	6.0
Suspected intoxication	No	254	90.4
	Yes	27	9.6
Posterior midline cervical spine tenderness	No	75	26.7
	Yes	109	38.8
Not done	97	34.5
45° active rotation	No	7	2.5
	Yes	14	5.0
Not done	260	92.5
Focal neurologic deficit	No	274	97.5
	Yes	7	2.5
CCR application	No	23	8.2
	Yes	258	91.8
NEXUS rule application	No	9	3.2
	Yes	272	96.8

CCR: Canadian C Spine Rule; NEXUS: The National Emergency X-Radiography Utilization Study

**Table 3:** Reason inadequacy of cervical spine radiography

	Patients	Frequency (%)
Absence of C7	113	64.6
Absence of more than C7 vertebrae	49	28.0
Absence of C1-C2 vertebrae	6	3.4
Absence of lateral view	1	0.6
Absence of open month view	14	8.0
Artifact	4	2.3

## Discussion

The present study revealed that the Nexus and the CCR rules are well applied in our emergency department. In a survey send to 61 Massachusetts emergency physicians, Weiner S et al reported that only 56% and 10% of them recognized using the NEXUS and CCR rules respectively in their current practices [7]. The most common reason cited for not using the NEXUS rules in this study was patient insistence on obtaining a radiograph. The most common reason for not using the CCR was that it is too difficult to remember and use in daily practice. The Canadian team have published controversial studies showing the superiority of CCR [3,8]. This debate between the NEXUS and the Canadian group may have decreased the reliably of physician to these rules [7]. Furthermore, Zoe et al in a meta-analysis of 15 studies demonstrated that despite their high sensitivity, these rules have low specificity [9]. In our department, physicians are encouraged to use at least one rule before sending patients to perform CSR.

The second finding of this current study is the low rate of adequate CSR. An adequate CSR includes three views: a true lateral view, which must include all seven cervical vertebrae as well as C7-T1 junction, an anterior posterior view and an open mouth odontoid view [10]. In the context of trauma these images are all difficult to acquire because the patient may be in pain, confused, unconscious, or unable to cooperate due to the immobilization devices. In 92% of case, the absence of lower cervical vertebrae was the reason of inadequacy of radiograph. Traction on the arms may facilitate visualization of all seven cervical vertebrae on the lateral film. The fact that our technologist is alone could explain such findings. In a retrospective study of 640 consecutive CSR, Gale et al reported entire cervical spine visualization in only 27.8% of patients [11]. The author concluded provocatively that CSR are inadequate to fully evaluate the cervical spine after blunt trauma, and supplemental computer tomography (CT) is commonly required. Several others studies have also suggested the superiority of CT in moderate to high-risk adults [12-14] as well as in lower risk context [15]. The systematic use of CT for initial evaluation of blunt cervical spine injury points out the problem of radiation exposure [16] and the unjustifiable raise of health care cost [13,14]. On the other hand, repeated attempts to obtain adequate CSR could also be ineffective. Moreover, the arrival of fasted low-dose radiation exposure CT, the raise of malpractice lawsuit procedure against emergency physicians and radiologist will probably decrease the number of CSR in the future [17]. The development and the increasing availability of low dose multidetector CT will be the solution [18].

## Conclusion

CCR and NEXUS are widely used in our emergency department. The high rate of inadequate CSR reinforces the debate about its utility in emergency condition.

### What is known about this topic

- Cervical blunt trauma is major health problem in developed countries;
- The Canadian C Spine Rule (CCR) and the National Emergency X-Radiography Utilization Study (Nexus) low criteria are well accepted as guide to help physician in case of cervical blunt trauma;
- While these rules are widely accepted, their current application and results are poorly studied.

### What this study adds

- The high rate of inadequate CSR reinforces the debate about its utility in emergency condition.

## Competing interests

The authors declare no competing interests.
